# MPTP-induced mouse model of Parkinson’s disease: A promising direction for therapeutic strategies

**DOI:** 10.17305/bjbms.2020.5181

**Published:** 2021-08

**Authors:** Musa Mustapha, Che Norma Mat Taib

**Affiliations:** 1 Department of Human Anatomy, Faculty of Medicine and Health Sciences, Universiti Putra Malaysia, Serdang, Selangor (Darul Ehsan), Malaysia; 2 Department of Human Anatomy, Faculty of Basic Sciences, College of Medical Sciences, Ahmadu Bello University, Zaria, Nigeria

**Keywords:** Parkinson disease, MPTP, C57BL mouse and MPTP-induced PD mouse

## Abstract

Among the popular animal models of Parkinson’s disease (PD) commonly used in research are those that employ neurotoxins, especially 1-methyl- 4-phenyl-1, 2, 3, 6-tetrahydropyridine (MPTP). This neurotoxin exerts it neurotoxicity by causing a barrage of insults, such as oxidative stress, mitochondrial apoptosis, inflammation, excitotoxicity, and formation of inclusion bodies acting singly and in concert, ultimately leading to dopaminergic neuronal damage in the substantia nigra pars compacta and striatum. The selective neurotoxicity induced by MPTP in the nigrostriatal dopaminergic neurons of the mouse brain has led to new perspectives on PD. For decades, the MPTP-induced mouse model of PD has been the gold standard in PD research even though it does not fully recapitulate PD symptomatology, but it does have the advantages of simplicity, practicability, affordability, and fewer ethical considerations and greater clinical correlation than those of other toxin models of PD. The model has rejuvenated PD research and opened new frontiers in the quest for more novel therapeutic and adjuvant agents for PD. Hence, this review summarizes the role of MPTP in producing Parkinson-like symptoms in mice and the experimental role of the MPTP-induced mouse model. We discussed recent developments of more promising PD therapeutics to enrich our existing knowledge about this neurotoxin using this model.

## INTRODUCTION

Parkinson’s disease (PD) is an insidiously progressive and irreversible neurodegenerative disease that mainly affects the older population [1]. Although the disease can appear at any age, the average age of onset is 60 years [2]. Garza-Ulloa [3] reported that PD is the second most popular neurodegenerative disease in the world after Alzheimer disease. PD is now the fastest growing neurological disorder and leading cause of disability globally with a total patient population from 1990 to 2016 of >6 million [4]. This number is expected to double to >12 million by 2040 [5]. Studies have shown that various factors, such as increasing life expectancy, increasing industrialization, and declining smoking rates, could increase the disease burden [6,7]. This rising disease incidence and prevalence globally make it a disease with huge economic, social, and public health importance [8].

Based on current thinking, two forms of PD exist: sporadic/late-onset and familial/early onset cases [9]. Epidemiological studies have reported that the familial form of PD only accounts for a few of the PD subjects, whereas the overwhelming majority of PD subjects have the sporadic type [10]. The etiology of PD is complex due to the heterogeneity of the disorder [11]. However, PD is believed to begin principally by degeneration of dopaminergic nigrostriatal neurons in the brain and secondarily by complex pathological mechanisms, including mitochondrial dysfunction, oxidative stress, apoptotic cell death, protein aggregation and misfolding, inflammation, excitotoxicity, loss of trophic factors, and other cell-death pathways [12]. PD patients present with a myriad of symptoms, including the four cardinal motor manifestations of tremor, rigidity, akinesia, and postural reflexes [13], as well as non-motor symptoms, such as dementia, anxiety, somnolence, urinary symptoms, attention deficit, hyposmia, and restless-leg syndrome, as the disease progresses [2,14]. The pathognomonic signs of PD are loss of dopaminergic neurons in the substantia nigra pars compacta (SNpc) and the formation of intraneuronal protein inclusions termed Lewy bodies (LBs), composed primarily of a-synuclein [15]. Jagmag et al. [16] also reported some neuronal losses in other parts of the brain, such as in the thalamic subnuclei and amygdala, serotoninergic neurons of the raphe nucleus, and the cholinergic nucleus basalis of Meynert, as the disease progresses.

Regardless of the tremendous advancement in the understanding of the disease mechanism, the presently approved PD treatments only provide limited therapeutic benefits [17]. This unmet clinical need to develop new therapeutic strategies has triggered further research to clarify the pathology of the disease. As a result, efforts have been made to emulate human PD by using animal models since studies have shown that they can mimic various aspects of PD features and thus support study of the disease pathophysiology and exploration of treatment possibilities [18].

The experimental animal models so far are of two main types: toxin models and genetic models. The transgenic models only simulate the familial form of PD, and the final neuropathological and behavioral features reminiscent of human PD are not fully recapitulated in this model [19]. The transgenic models also use transgenic technology, which makes them fairly expensive and thus not commonly used in PD research [19]. The common toxin models make use of some neurotoxins, such as paraquat, rotenone, 6-hydroxydopamine (6-OHDA), methamphetamine, and 1-methyl-4- phenyl-1,2,3,6-tetrahydropyridine (MPTP) to induce dopaminergic neurodegeneration in brains of animals [10,20,21]. The extent to which these neurotoxins phenocopy the salient features of PD and their related mechanisms varies greatly, especially in the sporadic type of PD [22]. Although PD-toxin models have played a significant role in defining critical disease-related mechanisms and have been at the forefront of evaluating novel therapeutic approaches, they also cannot fully mirror symptoms reminiscent of human PD [23].

However, for decades, mouse models using MPTP have been among the most extensively used in PD research because they have the advantage of easy practicality, affordability, and fewer ethical considerations and greater clinical correlation than those of other toxin models [10, 24]. Of note, the PD neurotoxic potential of MPTP in humans, monkeys, rodents, zebrafish, and *Caenorhabditis elegans* has also been documented [1, 25]. Although studies have shown that MPTP-intoxicated monkeys provide the best results for PD pathology, such as LB-like inclusions, the MPTP-mouse model is still more popular because of its practicality and feasibility [10, 16]. Currently, research involving the MPTP-induced mouse model of PD is in vogue and has been on the increase [18]. Among mice, different strains or the same strain from another source shows strikingly different sensitivity to the MPTP concerning the loss of DA neurons in the SNpc and striatum [10]. The mouse strain most sensitive to MPTP intoxication is C57BL/6, followed by CD-1, and BALB, with the least being Swiss Webster [26, 27]. Therefore, to obtain the best reproducible PD results from one experiment to another, male mice of ≥8 weeks of age, average weight of 22 g, and the same mouse strain must be obtained from the same source [28].

MPTP-mouse models of PD have provided more insight into the etiology and pathophysiology of this debilitating disease [29]. The importance in providing researchers with a unique model platform for testing the efficacy of novel neuroprotective drugs cannot be underestimated. Thus, in this review, the use of MPTP in mice to recapitulate PD symptoms will be highlighted, and the pharmacokinetics and pharmacodynamics of MPTP, MPTP administration dynamics, mechanisms of MPTP-induced neurotoxicity, and MPTP-induced PD mouse model laboratory findings to enrich our understanding of this neurotoxin will be discussed. To further promote the use of the MPTP-induced mouse model of PD by researchers for developing more promising therapeutic strategies, this review will also highlight its advantages and disadvantages, its experimental role, and recent developments in PD therapeutics using the model. Using the keywords of Parkinson’s disease, MPTP, C57BL/6 mice, and MPTP-induced PD mouse, all of the relevant literature used in this review was searched and collected from credible scientific databases, including Science Direct, Scopus, PubMed, and Google Scholar. Searches for laboratory findings from studies using the MPTP-induced mouse model of PD and for recent developments in PD therapeutics using this model were restricted to papers published from 2019 to date.

### Structure, pharmacokinetics, and pharmacodynamics of MPTP

**FIGURE 1:**
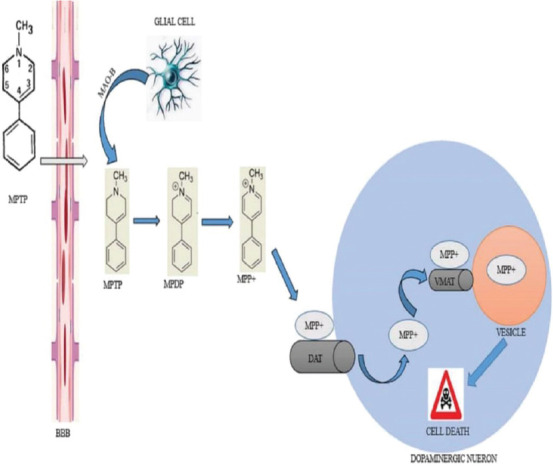
Structure, pharmacokinetics and pharmacodynamics of MPTP in the CNS. Upon injection of MPTP, it crosses the BBB and is converted to the toxic metabolite MPP+ by MAO-B. This metabolite is transported into the dopaminergic neuron by DAT. In the cytoplasm, MPP+ is further transported into vesicles by VMAT. Consequently, further concentration of MPP+ in the cytoplasm leads to a cascade of reactions that results in cell death. BBB: blood-brain barrier; CNS: central nervous system; DAT: dopamine transporter; MAO-B: monoamine oxidase type B; MPDP+: 1-methyl-4-phenyl-2, 3-dihydropyridine; MPP+: 1-methyl-4-phenylpyridinum; MPTP: 1-methyl-4- phenyl-1, 2, 3, 6-tetrahydropyridine; VMAT: vesicular monoamine transporter type 2.

MPTP is structurally a meperidine analog produced as a by-product in the process of synthesizing 1-methyl-4-phenyl-propionoxy-piperidine. Once this toxin is injected into the body of mice, it traverses the blood–brain barrier (BBB) into the central nervous system (CNS) with ease because of its lipophilicity [10]. In the CNS, monoamine oxidase type B (MAO-B) enzyme secreted by glial cells (astrocytes) converts MPTP to an intermediate metabolite, 1-methyl-4-phenyl-2,3-dihydropyridine (Figure 1), and subsequently to the final toxic metabolite, 1-methyl-4-phenylpyridinum (MPP+, Fig. 1) [30]. Cohen et al. [31] and Heikkila et al. [32] noticed striatal MPP+ depletion following treatment with MAO-B inhibitors, such as seleginine, which proved that inhibition of this enzyme significantly prevented formation of this toxic metabolite. MPP+, the active neurotoxin is a polar compound and as such, it cannot cross back through the BBB, indicating that it acts at the cellular level [28]. MPP+ selectively enters norepinephrine (NE) and dopaminergic (DA) neurons via the special transporters, NE transporter and DA transporter (DAT) respectively [33]. Studies by Takahashi et al. [34] proved that mice deficient in DAT were resistant to MPTP toxicity. Therefore, excessive expression of DAT will enhance MPTP neurotoxicity. Once in the NE/DA nerve cell, MPP+ forms a complex with neuromelanin in the axoplasm and is subsequently transported by vesicular monoamine transporter type 2 (VMAT-2) and stored in synaptosomal vesicles. This was confirmed by Gainetdinov et al. [35] in an experiment in which VMAT-2-deficient mice showed strikingly increased toxicity to MPTP. Therefore, selective toxicity of MPTP is directly related to the amount of DAT [36] and inversely to the amount of VMAT-2 [37]. MPP+ continues to accumulate in synaptosomal vesicles to a point when the threshold is surpassed and cell death of DA nigrostriatal neurons occurs in the SNpc and striatum (Figure 1).

### Mechanisms of MPTP-induced neurotoxicity

**FIGURE 2:**
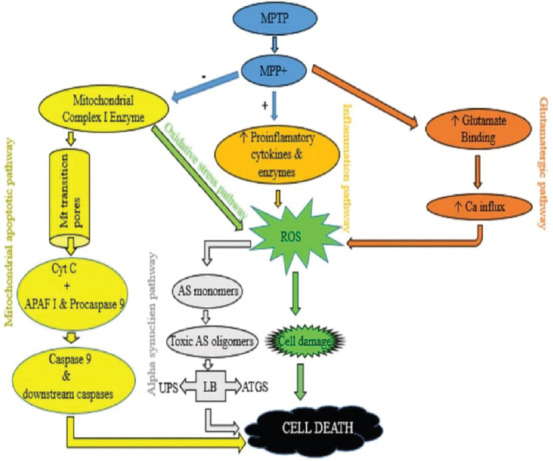
Summary of the neurotoxic pathways of MPTP. MPP+ causes inhibition of COMPLEX-1 in the mitochondria which leads to the opening of transitional pores and then release of cytochrome C which causes a cascade of reactions that leads to cell death (Mitochondrial apoptotic pathway). Inhibition of COMPLEX 1 also causes an increase in ROS which leads to cell damage and eventually cell death (Oxidative stress pathway). Further excessive production of ROS leads to formation of AS monomers, and the monomers then form toxic oligomers which then inhibits UPS and ATGS and eventually leads to cell death (Alpha synuclein pathway). MPP+ causes excessive binding of glutamate at the synaptic cleft. This causes Ca influx that leads to excessive production of ROS, which damages the cell and cell death occurs finally (Glutamatergic pathway). MPP+ activates microglia cell and induces release of proinflamatory cytokines/enzymes leading to excessive ROS production, then cell damage and eventually cell death (Inflammation pathway). APAF-1: apoptosis protease activating factor 1; AS: Alpha synuclien; ATGS: autophagy system; iNOS: Inducible nitric oxide synthase; LB: Lewy body; MPP+: 1-methyl-4-phenylpyridinium; MPTP: 1-methyl-4-phenyl-1,2,3,6-tetrahydropyridine; Mt: Mitochondria; ROS: Reactive oxygen species; UPS: ubiquitin-proteasome system; (-): downregulates; (+): upregulates; (↑): Activates.

Once the toxic metabolite of MPTP (MPP+) continues to accumulate and aggregates in synaptosomal vesicles of DA neurons, the amount eventually becomes too much in the cytoplasm and eventually triggers cell damage in the striatum and SNpc via the following pathways (Figure 2).

### Mitochondrial apoptotic pathway

MPP+ inhibits COMPLEX 1 in the mitochondria and induces less expression of anti-apoptotic proteins, such as Bcl2 [38, 39]. This inhibition hinders the electron transport chain and thus blocks ATP synthesis and increases reactive oxygen species (ROS) production leading to the opening of mitochondrial transition pores [28]. Cytochrome C is then released from a mitochondrion and forms a complex with pro-caspase-9 and apoptosis protease activating factor-1 [40]. The complex now formed activates caspase 9 and downstream caspases resulting in apoptosis and finally DA nigrostriatal cell death in the SNpc and striatum [41].

### Oxidative stress pathway

MPP+ inhibits nicotinamide adenine dinucleotide dehydrogenase in the mitochondria and allows excessive ROS production, such as H_2_O_2_, NO, and hydroxyl radicals [42, 43]. These ROS overwhelm the cellular antioxidant defense mechanism and cause DA nigrostriatal cell damage in the SNpc and striatum through lipid peroxidation, DNA damage, and protein cross-linkage [42, 44, 45].

### Alpha-synuclein pathway

Increases in ROS cause production of alpha-synuclein monomers [46]. As the levels of these monomers increase, they aggregate to form toxic alpha-synuclein oligomers. Oligomers of this kind can also be produced by mutation of the alpha-synuclein gene, *SNCA* [47, 48]. The oligomers inhibit the ubiquitin proteasome system (UPS) and autophagy system (ATGS), which are responsible for maintaining biochemical balance in the neuron [49, 50]. Failure of UPS leads to development of LBs, one of the pathological hallmarks of PD [28].

### Inflammation pathway

MPP+ triggers an inflammatory process characterized by T-cell infiltration into the striatum and SNpc with microglia activation [33, 51]. Activated microglia release proinflammatory factors, such as TNF-a, PGE2, IFN-g, and ROS, such as NO and H_2_O_2_, which are all toxic to neurons [52]. Nagarajan et al. [17] documented that activated microglia have an intrinsic role in MPTP-induced neurotoxicity because they upregulate inducible NO synthase and nicotinamide adenine dinucleotide oxidase. These two enzymes produce SO_4_^2−^ and NO, and being ROS, they cause oxidative stress and thus lead to death of DA nigrostriatal neurons in the SNpc and striatum [13].

### Glutamatergic pathway

MPP+ causes an increase in extracellular glutamate in the SNpc and striatum [53]. Glutamate binds to ionotrophic and metabotrophic receptors [52]. An increase in glutamate causes excessive and prolonged activity at the synaptic cleft, which causes an increase in the entry of ions, especially Ca2+ [33]. The influx of these ions increases the production of ROS, which leads to oxidative stress [44]. Also, an increase in glutamate can impair the function of the mitochondria resulting in a series of events that converts non-toxic levels of glutamate into higher cytotoxic levels [28].

### MPTP administration dynamics

**FIGURE 3:**
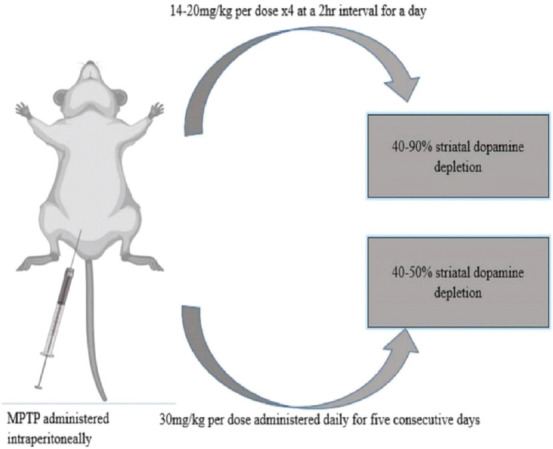
Schematic diagram of the commonest MPTP dosing regimen and route.

Different MPT-dosing regimens have been used by researchers to produce mouse-model features that closely mimic PD in humans [33]). The time course of any regimen used will determine the degree of apoptosis, striatal dopamine loss, and dopaminergic cell loss in the substantia nigra [54]. Compared with single administration, repeated administration of a particular regimen for a longer period produces more robust and irremediable neurodegeneration. Literature reports indicate that when more than one injection is given in 24 hours, it is called an acute administration regimen, whereas when a single injection is given daily for several consecutive or non-consecutive days or week, it is called a subacute or chronic administration regimen [13]. However, subacute and chronic regimens remain controversial because of the rapid toxicokinetics of the neurotoxin. For this reason, the most common regimen used irrespective of the aforementioned nomenclatures will be considered. The first of this regimen type involves a single MPTP injection for a total of four doses over 24 hours. In this regimen, striatal dopamine diminution can range from 40% (14 mg/kg per dose x4) to roughly 90% (20 mg/kg per dose x4) 7 days after the last MPTP dose depending on the doses given (Fig. 3) [54]. Another popular regimen was developed by Tatton and Kish [55]. Here, a single injection of MPTP free base 30 mg/kg is given daily for 5 consecutive days (Figure 3). In this method, 40%–50% striatal dopamine depletion (Fig. 3) and apoptosis is seen especially in young-adult C57/BL mice, and by day 21, the dopaminergic lesion stabilizes after administration of MPTP [54].

Regarding the administration site, many studies have agreed on the intraperitoneal route as ideal because several MPTP administered via this route remarkably impair motor function and induce DA neuronal damage [13,35]. There are conflicting reports regarding the appearance of Lewy body-like cytoplasmic inclusion when MPTP is administered intraperitoneally. To confirm this, Alvarez-Fischer et al. [56] and Shimoji et al. [57] found that 28-day chronic intraperitoneal MPTP administration (23 mg/kg/day), 7-day subacute intraperitoneal injection (20 mg/kg/day), and 28-day subcutaneous infusion (23 mg/kg/day) did not produce or trigger the formation of Lewy body neuronal inclusions. However, studies by Gibrat et al. [58] and Giráldez-Pérez et al. [59] demonstrated that chronic intraperitoneal infusion of MPTP (46 mg/kg/day) for 14 days with osmotic minipumps reproduced the formation of neuronal inclusions as observed by alpha-synuclein expression within the cytoplasm of dopaminergic neurons in the SNpc. It could be inferred that low-dose MPTP may not be adequate to facilitate formation of LBs. According to Jiang et al. [60], increased lactate levels in the brain are associated with formation of inclusion bodies simply because they can activate AMP-activated protein kinase and promote a-synuclein accumulation and phosphorylation.

### Laboratory findings in the MPTP-induced mouse model of PD

The MPTP neurotoxin is the gold standard for studying and understanding the processes involved in the DA nigrostriatal neuron death in PD [61]. Studies have used different MPTP-dosing regimens in mice over the years, and similar findings have been reported in virtually all of the studies; i.e., significant motor impairment and damage to the nigrostriatal DA pathway with marked loss of DA neurons in the SNpc and striatum [62, 63]. This explicit and reproducible neurotoxic effect on the nigrostriatal system is an exclusive asset of this model and is similar to the neurotoxic effect seen in PD patients [64]. It is important to note that LBs, which are one of the pathological hallmarks of PD, were not examined in the reported studies in this section probably because the model seldom shows this hallmark [21]. Since studies have shown MPTP to be selectively more toxic to the C57BL/6 mouse strain [28], only findings on this mouse strain will be reported in this section. Table 1 summarizes some laboratory findings in an MPTP-induced C57BL/6 mouse model of PD.

**TABLE 1:**
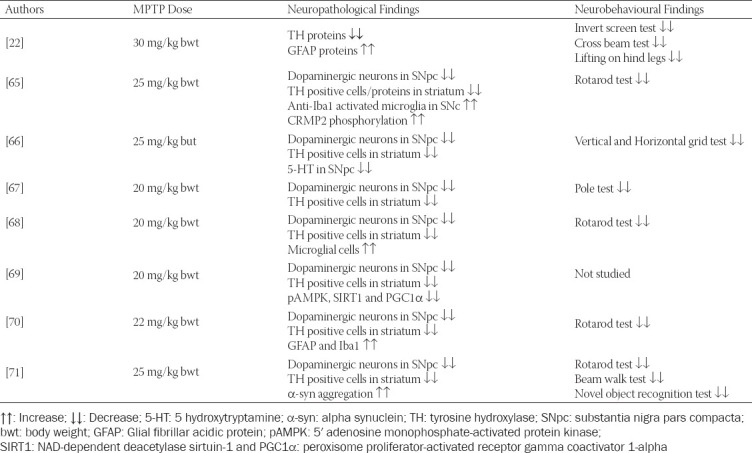
Laboratory findings in MPTP-induced C57BL/6 mouse model of PD

### Advantages of MPTP-induced mouse model of PD

1. The ability of the MPTP-mouse model to almost mirror the parkinsonian symptoms seen in human PD is the main reason for its usage [13,27].
2. Has helped to improve our knowledge of the molecular and cellular mechanism behind PD [18, 72, 73].
3. Is cheap, easy to handle, and has fewer ethical considerations than those of other toxin-induced animal models [19,74].
4. Reveals non-motor symptoms of PD [75].
5. In electrophysiological studies, Wallace et al. [76] reported the role of MPTP-mouse models in aiding deep brain stimulation-related therapy.
6. Has also helped in developing promising therapeutic strategies for neuroprotection and neurorestoration [77].
7. Has helped advance our understanding of the role played by mitochondrial dysfunction in PD [33, 78].
8. Has enhanced our knowledge of the role of autophagy in PD pathogenesis [79, 80].
9. According to Filograna et al. [81], the MPTP-mouse model has led to significant improvement in clinical research in PD.

### Disadvantages of MPTP-induced mouse model of PD

1. The most common drawback of this model is that it rarely induces Lewy body formation in most of the studies [19].
2. Behavioral features reminiscent of human PD is difficult to demonstrate in this model [82].

### Recent developments in therapeutics regarding PD using the MPTP-induced mouse model of PD

Efforts aimed at developing anti-parkinsonian therapeutics have shown encouraging results in a preclinical MPTP neurotoxic mouse model [23, 29, 65, 69, 83-85]. These therapeutics have proven their efficacy in aiding diagnosis and their ability to slow down, reverse, or actually prevent PD symptoms in this preclinical model. However, whether or not these preclinical findings can be translated into clinical trials is a huge question. The good news is that these therapeutics can stimulate translational research toward their neuroprotective adjuvant potentials in human PD. Table 2 summarizes the recent development in PD therapeutics using the MPTP-induced C57BL/6 mouse model of PD in 2019 and 2020.

**TABLE 2:**
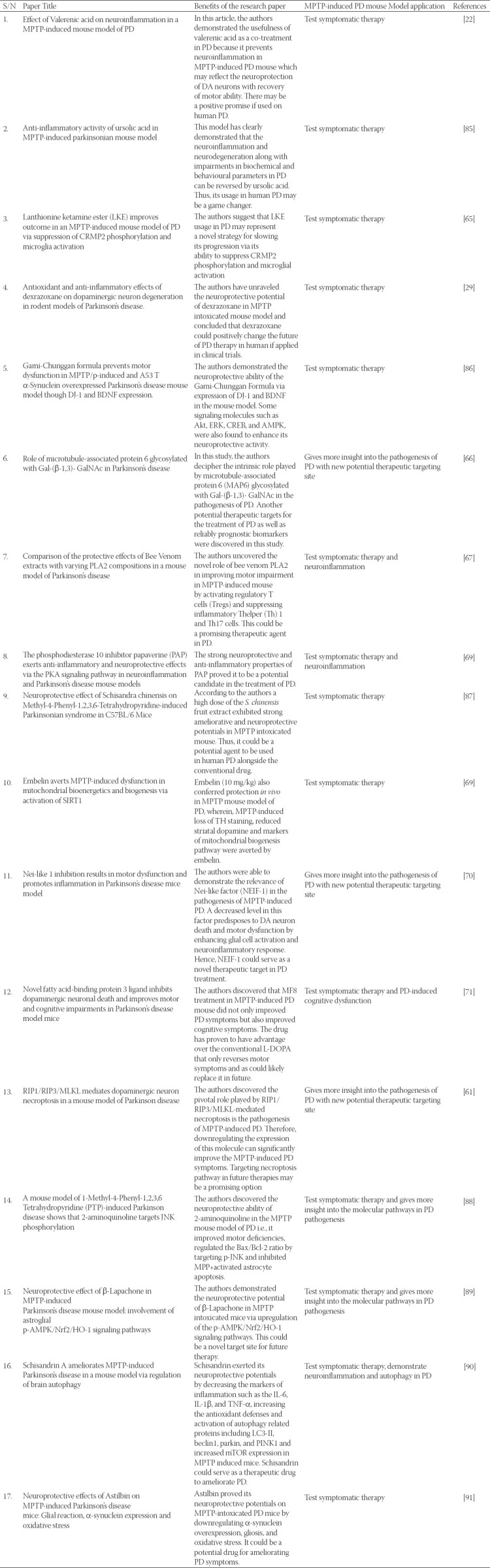
Recent development in PD therapeutics using MPTP-induced mouse model of PD.

## CONCLUSIONS

Transgenic and neurotoxin models have been used to mimic parkinsonian symptoms that are reminiscent of human PD. Although they all have the limitation of not fully mimicking PD symptoms, the MPTP-induced mouse model of PD now stands out among the other toxin models in PD research. This model is cheap to acquire, easy to handle, has fewer considerations than those of other toxin-induced animal models, is more practical, and shows good clinical correlation. Despite its shortcomings, this model has enhanced our understanding of the cellular and molecular mechanisms behind DA neuron death in the SNpc and striatum and provided researchers an avenue for exploring the neuroprotective and neurorestorative potentials of more novel therapeutic and adjuvant agents for PD. We believe that this model can further be perfected under the unrelenting efforts of researchers so that all of the pathological and phenotypical features reminiscent of human PD can be recapitulated. If a cocktail of miRNA or siRNA is introduced into the MPTP mouse-model system, a more robust and precise PD model showing all of the symptoms of human PD is likely to be obtained. Additionally, an improved model can be produced by combining the MPTP neurotoxin and genetic mouse models so that the progressive neurodegeneration associated with PD can fully be appreciated. Based on this premise, the MPTP-induced mouse model of PD may help researchers develop treatments that may allow PD patients to lead very close to normal lives.
